# The use of aluminum hydroxide as adjuvant modulates the antibody response to food allergens

**DOI:** 10.1186/2045-7022-5-S3-P100

**Published:** 2015-03-30

**Authors:** Katrine Lindholm Bøgh, Mie Scharff Andreasen, Charlotte Bernhard Madsen

**Affiliations:** 1 National Food Institute, Technical University of Denmark, Søborg, Denmark

## Background

Aluminum hydroxide (Al(OH)_3_) is a widely used adjuvant for induction of experimentally Th2 responses in animals and hence has been used for examination of food protein allergenicity. Further Al(OH)_3_ is used as an adjuvant for subcutaneous allergy vaccines. In the present study we investigated the impact of the use of Al(OH)_3_ on the antibody specificity to the cow's milk allergens β-lactoglobulin and α-lactalbumin.

## Methods

The cow's milk proteins were denatured by reduction and alkylation. Brown Norway rats were immunised intraperitoneally with either the native or the denatured form of the allergen with or without Al(OH)_3_ as adjuvant. The specific IgG1 and IgE antibody responses were analysed against the native as well as the denatured allergen by different ELISAs.

## Results

Denaturation of proteins will result in the unfolding of the native structure. Thus, while linear epitopes are maintained, there will be a consequential loss of conformational epitopes. Therefore examining the antibody reactivity against both the native and the denatured form of the proteins, will allow for examination of the relative proportions between antibodies raised against linear and conformational epitopes. This study showed that the native allergens had a greater sensitising capacity than their denatured counterpart. While native allergens primarily induced antibodies against conformational epitopes, the denatured allergens only induced antibodies against linear epitopes. However, most interestingly, this study showed that the use of Al(OH)_3_ modulated the antibody response, increasing the percentage of antibodies raised against linear epitopes.

## Conclusion

The study demonstrated that adsorption of proteins to Al(OH)_3_ changes the antibody specificity to the proteins. As this modulation of the antibody response was most pronounced for rats immunised with the native form of the allergen, we hypothesis that Al(OH)_3_ causes adsorption-induced changes in the protein structure, probably leading to an unfolding of the protein.

